# Exploring functionally related enzymes using radially distributed properties of active sites around the reacting points of bound ligands

**DOI:** 10.1186/1472-6807-12-5

**Published:** 2012-04-26

**Authors:** Keisuke Ueno, Katsuhiko Mineta, Kimihito Ito, Toshinori Endo

**Affiliations:** 1 Division of Bioinformatics, Hokkaido University Research Center for Zoonosis Control, North 20 West 10, Sapporo, Hokkaido, 001-0020, Japan; 2 Graduate School of Information Science and Technology, Hokkaido University, North 14 West 9, Sapporo, Hokkaido, 060-0814, Japan

## Abstract

**Background:**

Structural genomics approaches, particularly those solving the 3D structures of many proteins with unknown functions, have increased the desire for structure-based function predictions. However, prediction of enzyme function is difficult because one member of a superfamily may catalyze a different reaction than other members, whereas members of different superfamilies can catalyze the same reaction. In addition, conformational changes, mutations or the absence of a particular catalytic residue can prevent inference of the mechanism by which catalytic residues stabilize and promote the elementary reaction. A major hurdle for alignment-based methods for prediction of function is the absence (despite its importance) of a measure of similarity of the physicochemical properties of catalytic sites. To solve this problem, the physicochemical features radially distributed around catalytic sites should be considered in addition to structural and sequence similarities.

**Results:**

We showed that radial distribution functions (RDFs), which are associated with the local structural and physicochemical properties of catalytic active sites, are capable of clustering oxidoreductases and transferases by function. The catalytic sites of these enzymes were also characterized using the RDFs. The RDFs provided a measure of the similarity among the catalytic sites, detecting conformational changes caused by mutation of catalytic residues. Furthermore, the RDFs reinforced the classification of enzyme functions based on conventional sequence and structural alignments.

**Conclusions:**

Our results demonstrate that the application of RDFs provides advantages in the functional classification of enzymes by providing information about catalytic sites.

## Background

High-throughput methods for structural genomics have produced an increasing number of protein structures to be solved by X-ray crystallography. The abundance of protein structure information in the Protein Data Bank (PDB) has increased the need and desire for structure-based function prediction [1] and has contributed to structure-based drug design [2]. However, two problems remain regarding the prediction of enzyme function. First, proteins within a superfamily, which are usually expected to share the same catalytic properties, can catalyze different reactions. There are reports that enzymes with 98% sequence identity, such as melamine deaminase and atrazine chlorohydrolase, may catalyze different reactions [3]. Second, two enzymes belonging to different superfamilies or fold classes can catalyze almost identical reactions [4].

The function of a protein can be affected by a small number of residues in a localized region of its three-dimensional structure [5]. Moreover, the specific arrangement and conformation of these residues can be crucial to a protein’s function and may be strongly conserved during its evolution, even when the protein sequence and structure change significantly [5]. For example, it was reported that the positioning of the reactive region of a substrate with respect to a cofactor is generally conserved in flavoenzymes [6].

Two methods for the description of local structures have been developed for predicting enzymatic functions. First, in the element-based description of catalytic residues, the catalytic roles in an enzymatic reaction are defined as acid–base, stabilizer or modulator roles [7]. Some insight into enzymatic reactions can be gained using this method, but manual annotation is inherently required. In addition, it is often difficult to differentiate between the acid–base and stabilizer roles because most structures solved by X-ray crystallography provide no information about hydrogen atoms. The second method is based on descriptions of substructures within the local structures of enzymes [8-23]. Many approaches to analyze and compare local structures have been proposed. One group of algorithms, which includes the PINTS [8], ETA [9-11] and FLORA [12] algorithms, scans protein structural databases using pre-calculated or automatically generated templates. Another group includes algorithms that compare the substructural epitopes of proteins using geometric hashing [13-15]. Similarly, SiteEngine [16] uses the concept of pseudocenters [17] to define the properties of the corresponding surface. None of these approaches can characterize catalytic sites and create feature vectors, even though they assess the similarity between catalytic sites.

In this study, we examine the structures of oxidoreductases and transferases using radial distribution functions (RDFs) that encode radially distributed properties of active sites centered around the reacting points of bound ligands. Thus, element-based and substructure descriptions are integrated into the RDF, assuming that catalytic roles are restricted by distances and that different catalytic residues can play identical roles. Although the topological correlation vector method of Stahl *et al.*[18] and WaveGeoMap, developed by Kupas *et al.*[19], provide feature vectors related to enzyme cavities, these descriptions use patches of active sites, regardless of the orientation of the catalytic residues. Therefore, it is still unclear whether the orientation of active sites around a reacting point is related to enzymatic function and how much of the orientation is conserved. Our method provides a different view of enzymatic function by focusing on the physicochemical properties surrounding a reacting point found in enzyme cofactors.

## Results

### Characteristic physicochemical pattern of active sites

**Figure 1:**
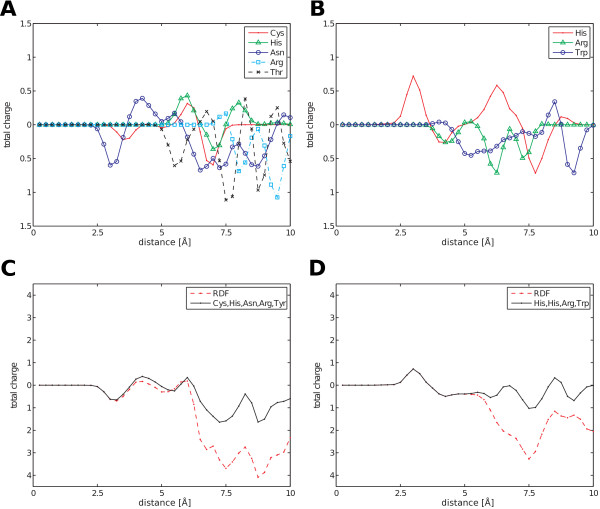
**RDF of total charge.**The line indicates the distances contributing to each catalytic residue peak for (**A**) the C4N atom of NAD in 1dc6 and (**B**) the FE atom of HEM in 1sog (PDB) and to each peak of degenerated catalytic residues and the RDFs for (**C**) 1dc6 and (**D**) 1sog (PDB).

To examine how catalytic residues contribute to the radially distributed properties of active sites, we decomposed the RDF into the total charge for each residue. Figure 1 shows the contributions made to the peaks and minima of the RDFs by the various catalytic residues surrounding the carbon atom (C4N) of the nicotinamide adenine dinucleotide (NAD) molecule in 1dc6 and the iron atom (FE) of the heme (HEM) molecule in 1sog (PDB). The first local minimum in 1dc6 corresponded to the nucleophilic cysteine residue and the asparagine residue that binds NAD (Figure 1A). The second local minimum and the last two minima were affected by the threonine residue that binds the substrate (Figure 1A). The peak at 6 Å corresponded to the histidine residue that activates the thiol group of the cysteine residue and to the cysteine residue itself (Figure 1A). The last two peaks were derived from the arginine residue that binds the substrate (Figure 1A). All of these five residues in 1dc6 are known to be critical for the enzymatic reaction. The first peak in 1sog corresponded to the proximal histidine residue (the heme axial ligand) and the distal histidine residue (the proton acceptor) (Figure 1B). The subsequent minimum at 7.5 Å was slightly affected by the histidine residues and the arginine residue (a transition state stabilizer) (Figure 1B). The small peak at 8.5 Å shown in Figure 1B was derived from the tryptophan residue (a radical intermediate). We can show that all of the four residues described in 1sog also play an important role in the catalytic reaction. Moreover, the degenerated total charge of the catalytic residues corresponded to the RDF with a range from 0 to 5 Å for both enzymes (Figure 1C, D). The bias of the RDFs toward a negative charge may be due to ignoring hydrogen atoms. These results show that catalytic residues are primarily responsible for the physicochemical properties of active sites.

Subsequently, to investigate whether the RDFs adequately discriminate between active sites, we selected pairs including the wild-type and a mutant form of the enzymes from the dataset. We then calculated the averaged Euclidean distances or cosine distances (1 minus the cosine similarity) of matched pairs (wild/wild or mutant/mutant) and mismatched pairs (wild/mutant) for each enzyme compared to the distance measure (the complement 100 minus the match score) obtained using SiteEngine (Table 1). As shown in Table 1, most of the pairs were agglomerated within a Euclidean distance of 222, and the RDFs were successful in revealing their similarity. The pairs from MDH_ECOLI were located approximately at a Euclidean distance of 322 from each other; however, two identical mutants were reported to have different conformations (PDB code: 1ib6 and 1ie3) [24]. Long distances were observed between the mismatched pairs compared to those of the matched pairs (Table 1). Of the mismatched pairs, the CCPR_YEAST proteins were particularly close to each other (< a Euclidean distance of 199), and the RDFs failed to identify their dissimilarity. However, the conformation of the catalytic site in the CCPR_YEAST protein is not altered by the mutation included in this analysis (PDB code: 3ccp) [25]. These results show that the Euclidean distance between the RDFs reflects the conformational changes in the active sites. The match scores from SiteEngine were similar to the distances between the RDFs. Although the distances between the RDFs were slightly poorer than the match scores in terms of the number of successful discriminations, the feature vector of the RDF is almost equivalent to the measure of SiteEngine in discriminating between the active sites. Thus, the active sites are characterized based on the physicochemical patterns of the RDFs.

**Table 1:** Effect of mutations on the physicochemical properties of active sites

UniProt	Ligand	RDF				SiteEngine	
		Euclidean		1 – cosine		100 – match score	
		w/w, m/m	w/m	w/w, m/m	w/m	w/w, m/m	w/m
CCPR_YEAST	HEM	198	195	0.0041	0.0041	**30.7**	**24.5**
CHOD_STRS0	FAD	**222**	**358**	**0.0015**	**0.0039**	51.9	59.4
FPRA_MYCTU	FAD	168	231	0.0039	0.0053	**30.7**	**56.6**
	NDP/ODP	228	340	0.0073	0.0147	**38.2**	**74.4**
FRDA_SHEFN	FAD	**219**	**605**	**0.0054**	**0.0400**	14.1	13.2
G3P_BACST	NAD	**131**	**164**	**0.0017**	**0.0023**	**23.1**	**26.9**
IDH_ECOLI	NAP	**370**	**369**	0.0357	0.0312	**19.5**	**55.8**
MDH_ECOLI	NAD	322	385	0.0023	0.0133	29.5	32.2
NIA1_MAIZE	FAD	163	300	0.0037	0.0132	25.0	37.2
OYE1_SACPS	FMN	201	224	0.0051	0.0064	34.3	40.3

The w/w and m/m columns show wild-type/wild-type or mutant/mutant pairs. The w/m columns show wild-type/mutant pairs. The results with statistically significant differences between the match and mismatch are shown in bold font. The statistical significance was assessed by Wilcoxon rank sum tests with a 5% significance level.

### Active site properties as the critical determinants of enzyme function

**Figure 2:**
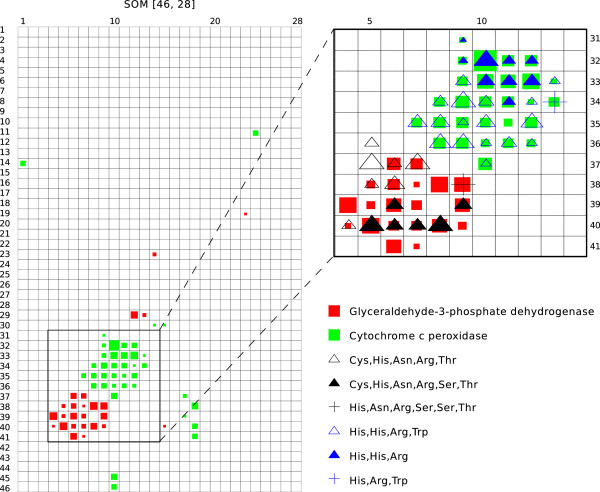
**Nonlinear projection of RDFs.** The SOM was run using an RDF with an Epanechnikov neighborhood function in a [46, 28]-sized rectangular lattice (left) and a magnified section (right). Following training, each node was colored according to the enzymes or catalytic residues in the RDFs that were mapped onto it. The size of the squares or triangles indicates the relative frequency of the mapped RDFs.

**Figure 3:**
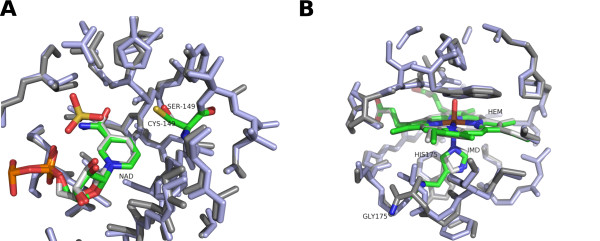
**Comparison between active sites in homologous enzymes mapped onto the SOM.** (**A**) Superposition of 3-phospho-glyceraldehyde dehydrogenase (PDB code 1dc6, node [38, 5]; and PDB code 1nq5, node [38, 9]). (**B**) Superposition of cytochrome c peroxidase (PDB code 1sog, node [36, 8]; and PDB code 1dso, node [34, 13]). The catalytic sites are indicated by light blue (1dc6, 1sog) and gray (1nq5, 1dso). The replaced residues are denoted and colored.

To investigate whether the RDFs account for a major part of the enzyme function, clustering of the RDFs was performed using a self-organizing map (SOM) approach. Figure 2 shows the results for glyceraldehyde-3-phosphate dehydrogenase (GAPDH) and cytochrome c peroxidase (CCP). The GAPDH residues were mainly distributed in the area around node [39, 6], including the two different catalytic sites (Figure 2). Within the GAPDH distribution, 1 dc6 from *Escherichia coli* and 1nq5 from *Bacillus stearothermophilus* (PDB) were closely positioned at nodes [38, 5] and [38, 9], respectively. The only difference between the catalytic sites in this orthologous pair is the replacement of cysteine 149 with serine leading to a 10^4^-fold reduction in dehydrogenase activity [26] (Figure 3A).

Similarly, the CCP residues were mainly localized in the area around node [33, 10], including the two different catalytic sites (Figure 2). Within the CCP distribution, 1sog and 1dso from *Saccharomyces cerevisiae* (PDB) were positioned at nodes [36, 8] and [34, 13], respectively. In the active site of 1dso, histidine 175 is replaced by glycine (Figure 3B). Thus, the results show that the obtained clusters of enzymes consist of clusters of their catalytic sites, suggesting that the RDFs of active sites account for a major part of the enzyme function.

### Prediction of enzyme functions based on the physicochemical properties of active sites

In this study, we sought to identify functionally related enzymes by clustering active sites. First, we utilized the EC number for assignment of RDFs to analyze the SOM clustering. An overview of the SOM is summarized in Additional files 1–4, for Additional file 1: Tables S1, Additional file 2: Table S2, Additional file 3: Figure S1, Additional file 4: Figure S2. Tables 2 and 3 show the division of the RDFs into nodes, each of which is labeled with its EC number. The partial RDFs labeled with the EC numbers indicated a well-defined segregation, discriminating among 76% of the EC numbers of oxidoreductases and among 55% of the EC numbers of transferases.

**Table 2:** SOM assignment of RDFs of oxidoreductases

Node composition	EC	SCOP^§^	Catalytic residues
Occupied by one class	2,929 (156)	1,966 (77)	949 (67)
Conflict*	129 (27)	42 (9)	112 (15)
All RDFs	4,092 (241)	2,526 (100)	1,910 (231)

The numbers indicate the RDF counts assigned to the nodes, and the number of classes is shown in parentheses. The SOM was performed by the RDF with an Epanechnikov neighborhood function in a [46, 28]-sized rectangular lattice

*One class is more than 80% of the total. ^§^The nodes were labeled using SCOP[44].

**Table 3:** SOM assignment of RDFs of transferases

Node composition	EC	SCOP^§^	Catalytic residues
Occupied by one class	885 (59)	526 (37)	356 (40)
Conflict*	25 (6)	12 (3)	11 (2)
All RDFs	1,444 (119)	797 (60)	736 (122)

The numbers indicate the RDF counts assigned to the nodes, and the number of classes is shown in parentheses. The SOM was performed by the RDF with a cut-Gaussian neighborhood function in a [40, 19]-sized rectangular lattice.

*One class is more than 80% of the total. ^§^The nodes were labeled using SCOP[44].

Then, to evaluate how many of the active sites are associated with enzyme functions, we performed a statistical analysis of the results of the SOM clustering. The averaged *F*-measure of all of the assigned EC numbers of oxidoreductases was 0.87, ranging from 0.22 to 1.00. Over 88% of the active sites of oxidoreductases were assigned to an EC number (see Additional file 5: Table S3). Similarly, the averaged *F*-measure of all of the assigned EC numbers of transferases was 0.88, ranging from 0.33 to 1.00. Over 88% of the active sites of transferases were assigned to an EC number (see Additional file 6: Table S4).

### Prediction performance in comparison with sequence and structural alignment-based annotation

To clarify the contribution of the RDFs to the functional annotation of the enzymes, we examined the relationship of the RDFs with different measures, such as sequence and structural alignment. First, we performed statistical analyses of these measures. Tables 4 and 5 show the partial correlation coefficients between the SOM distance, active site distance, local and global sequence similarities and structural similarity. The SOM distance among the RDFs was distinct from the other methods.

**Table 4:** Partial correlation between the different measures of oxidoreductases

Measures	MAMMOTH	Needleman- Wunsch	Smith- Waterman	Site Engine*	SOM distance
MAMMOTH		0.409	0.148	−0.318	−0.084
Needleman- Wunsch	0.409		0.404	−0.198	0.009
Smith- Waterman	−0.148	0.404		−0.101	−0.015
SiteEngine*	−0.318	−0.198	−0.101		0.052
SOM distance	−0.084	0.009	−0.015	0.052	

*The complement 100 minus the match score.

**Table 5:** Partial correlation between the different measures of transferases

Measures	MAMMOTH	Needleman- Wunsch	Smith- Waterman	Site Engine*	SOM distance
MAMMOTH		0.375	−0.020	−0.284	−0.078
Needleman- Wunsch	0.375		0.642	−0.309	−0.006
Smith- Waterman	−0.020	0.642		−0.142	−0.058
Site Engine*	−0.284	−0.309	−0.142		0.049
SOM distance	−0.078	−0.006	−0.058	0.049	

*The complement 100 minus the match score.

Next, the SOM distances among the RDFs were evaluated for their ability to annotate enzyme function in datasets that had not been correlated with known functions by either structural or sequence alignments due to pairwise identities below 25%. Tables 6 and 7 show the area under curve (AUC) values of the SOM distances for these datasets. In oxidoreductases, these values, ranging from 0.729 to 0.746, represented higher performance compared to the values obtained using sequence and structural alignments (Table 6). In transferases, the AUC values of 0.800 and 0.790 for the datasets with pairwise identities below 15% also represented higher performance compared to the values obtained using sequence and structural alignments (Table 7). These results showed that the SOM distance predicts enzyme function, even for enzymes with weak conventional similarities. Moreover, the SOM distance outperformed the match score of the SiteEngine based on substructure.

**Table 6:** Evaluation of the SOM distance with the RDFs for the prediction of enzyme function of oxidoreductases

Dataset*	AUC		
	SOM distance	SiteEngine	Alignment
MAMMOTH	0.746	0.410	0.415
Needleman-Wunsch	0.729	0.558	0.654
Smith-Waterman	0.744	0.541	0.471

*The datasets were created by culling the pairs with greater than 25% pairwise identity. The SOM was run using an RDF with an Epanechnikov neighborhood function in a [46, 28]-sized rectangular lattice.

**Table 7:** Evaluation of the SOM distance with the RDFs for the prediction of enzyme function of transferases

Dataset*	AUC		
	SOM distance	SiteEngine	Alignment
MAMMOTH	0.800	0.626	0.376
Needleman-Wunsch	0.790	0.678	0.474

*The datasets were created by culling the pairs with greater than 15% pairwise identity. The SOM was run using an RDF with a cut-Gaussian neighborhood function in a [40, 19]-sized rectangular lattice.

We then confirmed the ability of detecting enzymes with pairwise identities below 25%. While the ETA detected 63 oxidoreductases and 65 transferases, the numbers of enzymes assigned to the nodes within the SOM distance of 5 nodes were 454 of oxidoreductases and 387 of transferases, suggesting that the coverage of the SOM detection was higher than that of the ETA (Table 8).

**Table 8:** Identification of remote orthologs assigned to the same nodes in the SOM

PDB query	PDB target	EC number	Identity (%)	ETA
1j1wA	1xkdB	1.1.1.42	9.9	-
2aczA	1jryA	1.3.99.1	17.1	detected
1nekA	1jrxA	1.3.99.1	17.4	-
1nenA	1jrxA	1.3.99.1	17.4	-
1qjdA	2aczA	1.3.99.1	17.4	detected
1d4dA	2b76A	1.3.99.1	18	detected
1d4eA	1kfyM	1.3.99.1	18	-
1i2zA	1uh5A	1.3.1.9	21.4	-
2gsmA	2qpeA	1.9.3.1	21.4	-
1ocrA	2qpeA	1.9.3.1	22.6	-
1qleA	2qpeA	1.9.3.1	22.6	-
1ar1A	2qpeA	1.9.3.1	23	-
1qr6B	2dvmA	1.1.1.38	23.1	detected
2dvmA	1pjlE	1.1.1.38	23.1	-
1d1gA	1rb2A	1.5.1.3	24.9	-
1ra2A	1d1gA	1.5.1.3	24.9	-
1cm0A	1fy7A	2.3.1.48	9.8	-
1cm0A	1mj9A	2.3.1.48	10.6	-
2dpmA	1nw5A	2.1.1.720	13.6	-
1nw7A	2oreE	2.1.1.720	14	-
1gc3E	1oxoA	2.6.1.1	15.5	-
1gc3F	9aatA	2.6.1.1	15.5	-
1ahgA	1j32B	2.6.1.1	15.8	-
1akaA	1gc3F	2.6.1.1	16	-
3bo5A	1zkkB	2.1.1.430	17.5	-
1g55A	2qrvD	2.1.1.370	17.6	-
3pgtA	2caqA	2.5.1.18	19.2	-
2fyfA	1bjoA	2.6.1.52	19.6	-
1dl5B	1i1nA	2.1.1.770	20.5	-
1dl5B	1kr5A	2.1.1.770	20.5	-
1i1nA	1dl5A	2.1.1.770	20.5	-
1kr5A	1dl5A	2.1.1.770	20.5	detected
3aatA	1gc3H	2.6.1.1	22.5	-

### Structural genomics prediction

To perform a blind validation for proteins with unknown function, we used the SOMs trained by oxidoreductases and transferases to predict enzyme functions of 102 proteins in structural genomics. While the coverage of the ETA predictions was 31%, the SOM predictions covered 57% of the query structures (Table 9). Of the predicted EC numbers, the rates of validated prediction that the EC number is compatible with the bound ligands were 59% of the ETA predictions and 72% of the SOM predictions, suggesting the SOM predictions provide a clue to annotate these functions (Table 9).

**Table 9:** SOM predictions for the proteins with unknown function in structural genomics

PDB (Ligand)	SOM	ETA
1h2hA (NAD)	**1.3.1.26**	**1.4.1**, 4.3.1
1npdA (NAD)	1.14.99.3	**1.1.1**, 5.4.99
1o61A (PLP)	2.1.1.104	**2.6.1**, 6.3.4
1o8cA (NDP)	**1.1.1.2**	2.3.3, 5.4.4, 6.3.2
1rljA (FMN)	**1.8.1.2**	2.4.1
1t57A (FMN)	**1.8.1.9**	3.2.1
1ue8A (HEM)	1.2.1.9	1.14.14, 2.3.2, 2.7.7, 3.5.4, 3.6.1, 4.2.99, 5.1.3
1ve3A (SAM)	**2.1.1.104**	**2.1.1**, 3.1.3
1ve3B (SAM)	2.6.1.1	**2.1.1**, 3.1.3, 3.5.3, 5.1.3
1xq6A (NAP)	1.2.4.4	**1.6.5**
1y81A (COA)	**2.3.1.85**	1.13.11, 2.3.2, 2.7.10, 2.8.1, 3.6.1, 3.6.3, 4.1.2, 4.3.1, 6.3.2
1yoaA (FAD)	1.3.1.24 1.5.1.30	1.3.1, **1.6.8**, 2.7.4, 3.4.21, 3.7.1
1yreD (COA)	2.1.1.79	1.1.1, 2.3.1, 3.4.11, 3.4.22, 4.2.99
2e6uX (COA)	2.5.1.18	3.5.1
2eisA (COA)	2.5.1.6	3.1.2
2gluA (SAM)	2.3.1.168	**2.1.1**, 3.4.24
2gqfA (FAD)	1.3.1.26	1.1.1, 1.18.6, 1.3.3, 1.7.1, 2.7.1, 2.7.7, 3.2.1, 3.3.2, 3.4.21, 4.1.1, 6.3.3, 6.3.5
2gswA (FMN)	**1.18.1.2**	1.5.1, 1.7.1, 3.1.4
2ptfA (FMN)	**1.8.1.7**	**1.14.13**
2q46A (NAP)	1.2.4.4	**1.6.5**
3cgvA (FAD)	**1.14.14.1**	2.4.1, 6.1.1
3dmeB (FAD)	**1.18.1.2**	3.5.2
3f2vA (FMN)	**1.6.5.2, 1.6.99.2**	1.10.99

The EC numbers compatible with the bound ligands are shown in bold font.

## Discussion

**Figure 4:**
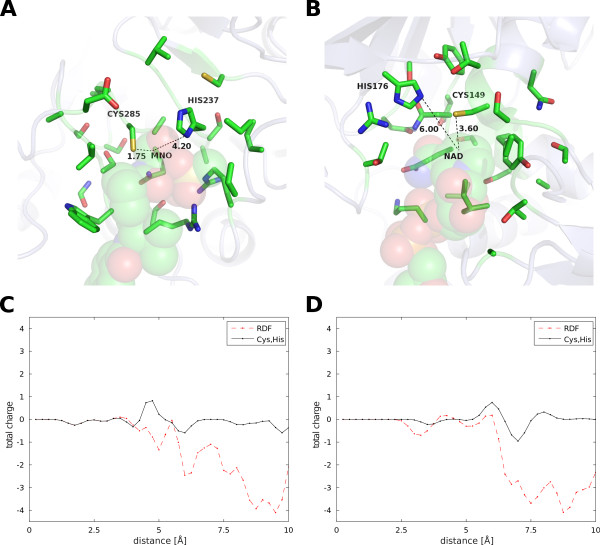
**Comparison between active sites in caspase-1 and 3-phospho-glyceraldehyde dehydrogenase.** Structures of active sites in (**A**) caspase-1 (PDB code 1bmq) and (**B**) 3-phospho-glyceraldehyde dehydrogenase (PDB code 1dc6) are drawn in stick representation. Comparison of the RDFs of the total charge for (**C**) 1bmq and (**D**) 1dc6, where the line indicates the distances contributing to each peak of the Cys-His catalytic diad and the RDFs for the C27 atom of MNO in 1bmq and the C4N atom of NAD in 1dc6.

Without using any templates, the RDFs centered around active sites are capable of clustering oxidoreductases and transferases based on their function. In this study, we applied our method only to the oxidoreductase and transferases classes of proteins. We focused on these classes for the following reasons. First, oxidoreductases exhibit a great variety of catalytic sites compared to other known classes, possibly because the redox potential is modulated by oxidoreductases. Second, the reaction centers are well-defined in oxidoreductases and transferases, consisting of a substrate and cofactor that mechanistically exchange electrons and protons. The catalytic residues are generally capable of assisting in the migration of protons from the reaction center, a role that strongly resembles the roles of other enzyme classes. For example, caspase-1 is a hydrolase that catalyzes the hydrolytic reaction of peptides; the cysteine residue nucleophilically attacks the substrate, which is followed by protonation of the histidine [27]. This mechanism closely resembles the catalytic behavior observed for GAPDH, an oxidoreductase [28]. Figure 4 shows the pattern of the Cys-His catalytic diad in 1bmq was similar to that in 1dc6. The peak shift may be due to the different position between substrate and cofactor. These similarities suggest that our method can be applied to other enzymes to predict additional protein functions. To apply our method to other enzymes, the reaction centers will require manual annotation. Reaction pairs published by KEGG RPAIR [29] that include candidate reaction centers are available for other enzymes and can be used for this purpose.

Understanding the orientation of catalytic sites is important for drug design. For a given G protein-coupled receptor, there are several types of ligands, classified as conformational change inducers, agonists, antagonists and inverse agonists [30]. The RDFs describe the orientation of catalytic sites, detecting conformational changes as well as enzyme function (Table 1). In addition, the description of the microenvironment produced by the RDF is better than simple superposition of catalytic sites when a particular functional group is not present (Figure 3).

In structural genomics, the RDFs would be advantageous for finding remote orthologs, especially when evolutionary pressure has enhanced sequence/structural divergence. Although sequence-based methods are the first choice for functional annotation, proteins with sequence identities of < 20-35% are problematic [31]. Measuring structural similarity is more informative for enzyme functions exhibiting distant relationships and/or convergent evolution. However, proteins within well-known superfamilies sharing the same structural topology, such as TIM barrels, do not always have the same functions [32]. In these cases, the measure of structural similarity alone does not correspond to functional similarity. Therefore, a specific measure representing functionality is desirable. We focused specifically on the local features around the catalytic site. Compared to the structural alignment, the functional annotation was reinforced by focusing on the reaction center (Tables 6 and 7). It is also likely that convergent evolution of an enzyme function depend less on evolutional process than on physicochemical properties of active sites (Tables 8 and 9). For proteins with unknown function, 41% of query structures were newly classified into the EC numbers (Table 9). However, the true performance of our method will be evaluated by revealing the actual function of those proteins. The combination of results obtained using different approaches will also improve the accuracy of function predictions.

## Conclusion

We propose a novel classification method for the prediction of enzymatic function based on the physicochemical properties of catalytic sites. The RDFs for predicting enzymatic functions are thus far limited to enzymes with bound ligands. For ligand-unbound structures, either homology modeling or superposition based on ligand-bound structures can be applied to our method. Our results suggest that the RDF provides a different perspective compared to structural and sequence alignments by focusing on a local feature because catalytic sites are thought to be more highly conserved than the overall sequences or structures of enzymes.

## Methods

### Dataset of active sites

Two sets of 1,880 oxidoreductase (EC1) and 789 transferase (EC2) protein structures were initially obtained from the PDB. In the case of NMR data, we used the first model in the PDB file. To simplify the filtering of the candidate active sites, structures including at least one cofactor or analogous compound were manually selected based on the annotation of PDBsum [33]. In this study, we used the substructures within 10 Å from the reaction centers of these cofactors as active site data. The reaction centers [34] of the cofactors are extensionally defined as follows: (1) atoms associated with bond formation and cleavage; (2) atoms exhibiting a change in charge; and (3) corresponding atoms in analogous compounds (see Additional files 1 and 2, Additional file 1: Tables S1, Additional file 2: Table S2). In oxidoreductases, a cofactor generally forms a part of the reaction center, acting as a donor and acceptor. Finally, based on this definition, 4,092 oxidoreductase and 1,444 transferase active sites corresponding to reaction centers were obtained. The subsequent encoding for comparison of active sites also used the Cartesian coordinates of these reaction centers as a starting point. In addition, a set of 102 protein structures with the key words of “structural genomics” and “unknown function” in the PDB was used for a blind validation of function prediction.

### Characterization of physicochemical properties of active sites

The values of physicochemical atomic properties, including the main chain of the amino acid residues, were empirically calculated by the PETRA server [35,36]. The atomic properties included were the total charge for electrostatic interactions and σ-electronegativity, π-electronegativity and effective atom polarizability for van der Waals interactions. These properties are based on the Partial Equalization of Orbital Electronegativities (PEOE) [35], which is independent of 3D structures. Because the side chains of proteins show various conformations, PEOE is suitable for describing their properties.

### Physicochemical encoding of active sites for the RDFs

The RDFs integrate the Gaussian distributions proportional to a physicochemical property at a distance from a starting point. Encoding of the RDF was performed by the method of Aires-de-Sousa *et al.*[37] with slight modification, as described below. The RDF as a function of the distance, *r,* is given by the following equation:

(1) $$g(r)=\sum_{i=1}^{N}p_{i}\cdot \frac{1}{\sqrt{2\pi }\sigma_{i}}\text{exp}\left(-\frac{{\left(r-r_{i}\right)}^{2}}{2\sigma_{i}{}^{2}}\right)$$

where *N* is the number of atoms in the active site residues; *r*_*i*_ is a constant for the inter-atomic distance between atom *i* and the reaction center atom (see Additional file 1: Table S1); *σ*^*2*^ is the fluctuation of the atoms around their averaged positions; and *p* is an atomic property (see Additional file 7: Figure S3). Thus, the RDFs naturally combine active site structures and their physicochemical properties, which exhibit an isotropic and rotationally invariant nature. In addition, we tested the effect of large *σ*^*2*^ in the RDFs to investigate the robustness to conformational change, suggesting that the RDFs were robust over a large range of *B*-factor (= 8π^2^*σ*^2^/3) in the PDB (see Additional file 8: Figure S4).

### SOM clustering and SOM distance

SOMs provide a topology-preserving map using a nonlinear projection of high-dimensional data onto a low-dimensional grid [38]. The low-dimensional grid is composed of nodes that represent data clusters. The neighboring nodes are connected to each other in the sense that they receive similar updates. Hence, SOMs provide information on the similarity between nodes. The SOM was run using a batch algorithm with an Epanechnikov or cut-Gaussian neighborhood function and an initial update radius of 5 or 10 nodes via implementation in the SOM Toolbox for Matlab (Mathworks, Inc.), which was developed in the Laboratory of Computer and Information Science of the Helsinki University of Technology.

In addition to the clustering, we also defined the SOM distance, which is the Euclidean distance between the SOM locations of the nodes on the grid, to obtain the distance measure between the active sites encoded by the RDFs.

### Software for the alignment of sequences, structures and active sites for comparative experiments

The sequences and structures were aligned using the Smith-Waterman algorithm [39] or the Needleman-Wunsch algorithm [40], both of which are implemented in the EMBOSS program package [41], or the structure-based alignment algorithms in the MAMMOTH program package [42]. All of the pairwise alignments were performed with the default parameters. The active sites were compared using a geometric hashing algorithm implemented in SiteEngine [16].

### Evaluation of SOM clustering

The *F*-measure is defined as a harmonic mean of both precision and recall that measures the extent to which a cluster contains only enzymes of particular EC classes and all enzymes of that EC class. A cluster was defined as all nodes labeled by an identical EC class. For a particular node in the SOM, we can calculate the centroid by finding the arithmetic mean of all of the RDFs. If an RDF in the centroid vector has a high value, then the corresponding EC class occurs frequently within the node. These EC classes can be used as labels for the node. The *F*-measure of a cluster with respect to an EC class was defined by the following equation:

(2) $$F=\frac{2\times precision\times recall}{precision+recall}$$

The precision of a cluster with respect to an EC class was defined as follows:

(3) $$precision=\frac{m}{M}$$

where *M* is the number of enzymes in a specific cluster, and *m* is the number of enzymes of the specified EC class in the cluster. Recall is the extent to which a cluster contains all of the enzymes of a specified EC class. The recall of a cluster with respect to an EC class was defined as follows:

(4) $$recall=\frac{m}{N}$$

where *N* is the number of enzymes in the EC class. The averaged *F*-measure for the validation of the classification performance was obtained by calculating the average of all of the EC classes, with 1 being the best value and 0 being the worst value.

### Evaluation of the measures for predicting enzyme functions

To estimate the degree of separation between two different functions when using a certain pairwise measure, analysis of a receiver operating characteristic (ROC) curve for the SOM distance was performed as well as local, global and structural alignments. Based on the cutoffs that determine whether the protein-protein pairs are predicted to be involved in the same function, i.e., true (match) or false (mismatch), the data are divided into true positives (*TP*), false positives (*FP*), false negatives (*FN*), and true negatives (*TN*). The true positive rate (*TPR*) and false positive rate (*FPR*) are defined as follows:

(5) $$TPR=\frac{TP}{TP+FN}$$

and

(6) $$FPR=\frac{FP}{FP+TN}$$

The ROC curve is a graphical plot of *TPR* versus *FPR*, showing the fidelity of discrimination at varying thresholds. The AUC is defined as the area under the ROC curve, representing the overall performance of discrimination. In this study, the SOM distances represented the dissimilarities among the RDFs. In the alignments, the similarities were the percentages of the number of aligned residues in the shortest protein.

### Partial correlation coefficients between the measures

To remove the influence of another variable from the Pearson correlation, the partial correlation coefficients between the measures were calculated from the correlation matrix, *Σ*. First, we computed the inverse matrix *Σ*^*-1*^ = (*π*_*ij*_) of the correlation matrix. Then, the partial correlation *θ*_*ij*_ between the measures *i* and *j* was defined by the following equation:

(7) $$\theta_{\mathrm{ij}}=-\frac{\pi_{\mathrm{ij}}}{\sqrt{\pi_{\mathrm{ii}}}\sqrt{\pi_{\mathrm{jj}}}}$$

In this study, we used the pseudo-inverse of the correlation matrix in the first step [43].

## Competing interests

The authors declare that they have no competing interests.

## Authors' contributions

KU wrote the code for the analysis, performed the experiments and wrote the manuscript. KM and TE participated in the design of the study and collaborated in writing the manuscript. KI was involved in revising manuscript. All of the authors have read and approved the manuscript.

## Supplementary Material

Additional file 1 **Table S1. Descriptions of the oxidoreductases mapped onto the SOM.** File “TableS1.xls” contains results of the SOM of oxidoreductases. The 4,092 RDFs were mapped onto a [46, 28]-sized rectangular lattice. The columns include a map position of a PDB code with functional annotation. Click here for file

Additional file 2 **Table S2. Descriptions of the transferases mapped onto the SOM.** File “TableS2.xls” contains results of the SOM of transferases. The 1,444 RDFs were mapped onto a [40, 19]-sized rectangular lattice. The columns include a map position of a PDB code with functional annotation. Click here for file

Additional file 3 **Figure S1. The SOM labeled with the EC numbers of oxidoreductases.** File “FigS1.pdf” contains results of the SOM of oxidoreductases. The 4,092 RDFs were mapped onto a [46, 28]-sized rectangular lattice, where each color of the node shows the major EC number in a node. The details of catalytic sites mapped onto the SOM were described in Table S1. Click here for file

Additional file 4 **Figure S2. The SOM labeled with the EC numbers of transferases.** File “FigS2.pdf” contains results of the SOM of transferases. The 1,444 RDFs were mapped onto a [40, 19]-sized rectangular lattice, where each color of the node shows the major EC number in a node. The details of catalytic sites mapped onto the SOM were described in Table S2. Click here for file

Additional file 5 **Table S3. Confusion matrix of the SOM for the EC numbers of oxidoreductases.** File “TableS3.xls” contains the confusion matrix of the SOM for the EC numbers of oxidoreductases. Each column of the matrix shows the number of RDFs in the assigned EC number, and rows represent the oxidoreductase list of the actual EC numbers. Click here for file

Additional file 6 **Table S4. Confusion matrix of the SOM for the EC numbers of transferases.** File “TableS4.xls” contains the confusion matrix of the SOM for the EC numbers of transferases. Each column of the matrix shows the number of RDFs in the assigned EC number, and rows represent the transferase list of the actual EC numbers. Click here for file

Additional file 7 **Figure S3. An example of an RDF.** File “FigS3.pdf” contains an example of an RDF for the total charge, σ-electronegativity, π-electronegativity and effective atom polarizability, which constitute a 160-dimensional variable as a feature vector. Click here for file

Additional file 8 **Figure S4. Robustness of functional classification to conformational change.** File “FigS4.pdf” contains the performance of the SOM clustering for the EC numbers as a *B*-factor in the RDFs is varied. The large *B*-factor in the RDFs corresponds to conformational change. The *F*-measure indicates the robustness of the classification performance. Click here for file
