# Supplementary figures and images for: Exploring functionally related enzymes using radially distributed properties of active sites around the reacting points of bound ligands
Source: BMC Struct Biol. 2012 Apr 26;12:5. doi: 10.1186/1472-6807-12-5 (PMC3408369; doi:10.1186/1472-6807-12-5)

## SOM [46, 28]

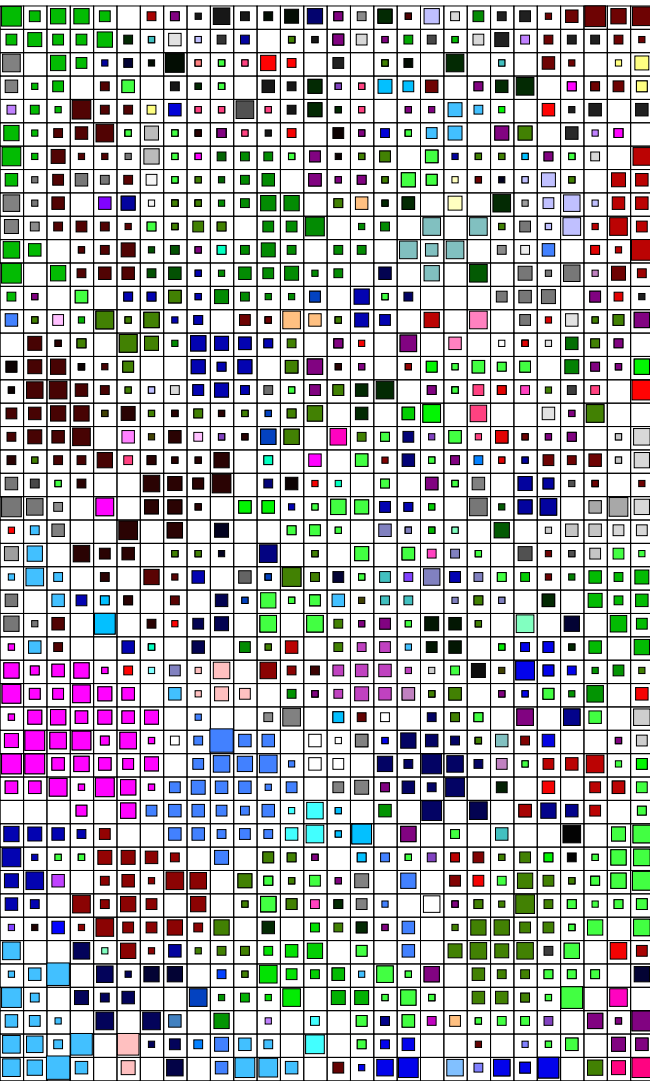

1. . .  
 1.1.1.1  
 1.1.1.10  
 1.1.1.100  
 1.1.1.130  
 1.1.1.133  
 1.1.1.138  
 1.1.1.14  
 1.1.1.141  
 1.1.1.146  
 1.1.1.149[1.1.1.189  
 1.1.1.149[1.3.1.20  
 1.1.1.153  
 1.1.1.158  
 1.1.1.159  
 1.1.1.178[1.1.1.35  
 1.1.1.184  
 1.1.1.184[1.1.1.189][1.1.1.197  
 1.1.1.188[1.1.1.213][1.1.1.63][1.1.1.64][1.3.1.20  
 1.1.1.193  
 1.1.1.193[3.5.4.26  
 1.1.1.195  
 1.1.1.1[1.1.1.284  
 1.1.1.2  
 1.1.1.204  
 1.1.1.205  
 1.1.1.206  
 1.1.1.21  
 1.1.1.213  
 1.1.1.213[1.3.1.20  
 1.1.1.219  
 1.1.1.22  
 1.1.1.225[1.1.1.50  
 1.1.1.23  
 1.1.1.236  
 1.1.1.25  
 1.1.1.252  
 1.1.1.25[2.5.1.19][2.7.1.71][4.2.1.10][4.2.3.4  
 1.1.1.26  
 1.1.1.267  
 1.1.1.268  
 1.1.1.27  
 1.1.1.271  
 1.1.1.272  
 1.1.1.274  
 1.1.1.28  
 1.1.1.282  
 1.1.1.290  
 1.1.1.3  
 1.1.1.30  
 1.1.1.31  
 1.1.1.35

1.1.1.35[4.2.1.107  
 1.1.1.35[4.2.1.17][5.1.2.3][5.3.3.8  
 1.1.1.37  
 1.1.1.37[1.1.1.82  
 1.1.1.38  
 1.1.1.40  
 1.1.1.42  
 1.1.1.44  
 1.1.1.47  
 1.1.1.49  
 1.1.1.5  
 1.1.1.50  
 1.1.1.53  
 1.1.1.6  
 1.1.1.62  
 1.1.1.67  
 1.1.1.77  
 1.1.1.8  
 1.1.1.82  
 1.1.1.85  
 1.1.1.95  
 1.1.2.3  
 1.1.3.10  
 1.1.3.15  
 1.1.3.21  
 1.1.3.38  
 1.1.3.4  
 1.1.3.41  
 1.1.3.6  
 1.1.5.2  
 1.1.99.18  
 1.1.99.28  
 1.1.99.31  
 1.1.99.8  
 1.10.2.2  
 1.11.1.1  
 1.11.1.10  
 1.11.1.11  
 1.11.1.13  
 1.11.1.5  
 1.11.1.6  
 1.11.1.7  
 1.13.11.52

1.13.12.4  
 1.14.11.16  
 1.14.11.17  
 1.14.11.18  
 1.14.11.19  
 1.14.11.21  
 1.14.12.17  
 1.14.13.2  
 1.14.13.25  
 1.14.13.3  
 1.14.13.39  
 1.14.13.48[1.14.13.49][1.14.13.80  
 1.14.13.67[1.14.13.97  
 1.14.13.7  
 1.14.13.70  
 1.14.13.92  
 1.14.14.1  
 1.14.14.1[1.6.2.4  
 1.14.15.  
 1.14.15.1  
 1.14.99.3  
 1.17.1.4[1.17.3.2  
 1.17.99.1  
 1.18.1.2  
 1.18.1.3  
 1.2.1.11  
 1.2.1.12  
 1.2.1.13  
 1.2.1.19  
 1.2.1.2  
 1.2.1.27  
 1.2.1.3  
 1.2.1.38  
 1.2.1.3[1.2.1.8  
 1.2.1.4  
 1.2.1.5  
 1.2.1.59

1.2.1.9  
 1.2.3.3  
 1.2.4.4  
 1.2.99.2  
 1.3.1.10[1.3.1.38  
 1.3.1.2  
 1.3.1.24  
 1.3.1.24[1.5.1.30  
 1.3.1.26  
 1.3.1.34  
 1.3.1.42  
 1.3.1.43  
 1.3.1.48[1.3.1.74  
 1.3.1.56  
 1.3.1.74  
 1.3.1.9  
 1.3.3.1  
 1.3.3.11  
 1.3.3.4  
 1.3.3.6  
 1.3.99.1  
 1.3.99.10  
 1.3.99.2  
 1.3.99.20  
 1.3.99.3  
 1.3.99.7  
 1.4.1.1  
 1.4.1.13  
 1.4.1.16  
 1.4.1.17[1.5.1.1][1.5.1.21  
 1.4.1.20  
 1.4.1.21  
 1.4.1.3  
 1.4.3.16  
 1.4.3.19  
 1.4.3.2  
 1.4.3.3

1.4.3.4  
 1.4.3.5  
 1.4.7.1  
 1.5.1.10  
 1.5.1.12  
 1.5.1.12[1.5.99.8  
 1.5.1.2  
 1.5.1.20  
 1.5.1.3  
 1.5.1.33  
 1.5.1.34  
 1.5.1.3[2.1.1.45  
 1.5.1.5[3.5.4.9][6.3.4.3  
 1.5.1.5[3.5.4.9][6.3.4.3  
 1.5.1.6  
 1.5.3.1  
 1.5.3.11  
 1.5.5.1  
 1.5.8.2  
 1.5.99.12  
 1.6.1.2  
 1.6.2.4  
 1.6.5.  
 1.6.5.2[1.6.99.2  
 1.6.5.5  
 1.6.8.  
 1.6.99.1  
 1.6.99.3  
 1.6.99.5  
 1.7.1.1  
 1.7.2.1[1.7.99.1  
 1.7.2.2  
 1.7.3.4  
 1.7.7.1  
 1.7.99.4  
 1.8.1.12  
 1.8.1.14

1.8.1.2  
 1.8.1.4  
 1.8.1.5  
 1.8.1.7  
 1.8.1.9  
 1.8.3.1  
 1.8.7.1  
 1.9.3.1

Supplement: Additional file 3 — Figure S1. The SOM labeled with the EC numbers of oxidoreductases. File “FigS1.pdf” contains results of the SOM of oxidoreductases. The 4,092 RDFs were mapped onto a [46, 28]-sized rectangular lattice, where each color of the node shows the major EC number in a node. The details of catalytic sites mapped onto the SOM were described in Table S1. [file 1472-6807-12-5-S3.pdf]

# SOM [40, 19]

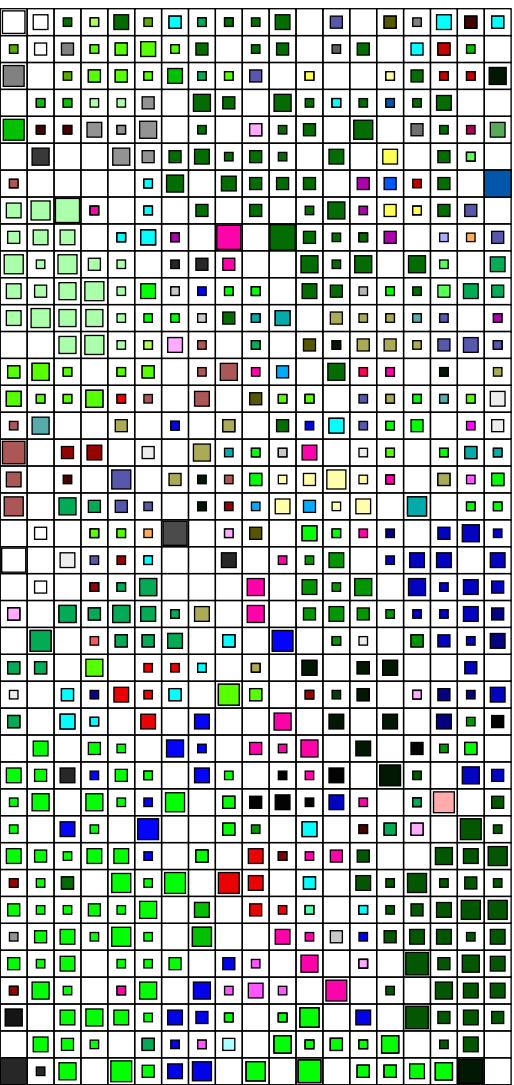

- 1.2.4.1
- 1.3.1.76|2.1.1.107|4.99.1.4
- 2.1.1.1.1
- 2.1.1.1.104
- 2.1.1.1.107
- 2.1.1.1.125
- 2.1.1.1.125,2.1.1.-
- 2.1.1.1.127
- 2.1.1.1.130
- 2.1.1.1.150
- 2.1.1.2
- 2.1.1.2.0
- 2.1.1.2.8
- 2.1.1.3.1
- 2.1.1.3.2
- 2.1.1.3.4
- 2.1.1.3.7
- 2.1.1.4.3
- 2.1.1.4.5
- 2.1.1.4.8
- 2.1.1.4.9
- 2.1.1.5.1
- 2.1.1.5.6
- 2.1.1.5.7
- 2.1.1.6
- 2.1.1.6.7
- 2.1.1.6.8
- 2.1.1.7.2
- 2.1.1.7.7
- 2.1.1.7.9
- 2.1.1.8
- 2.1.1.8.0
- 2.1.1.9.8
- 2.1.2.1
- 2.1.2.1.0
- 2.1.3.1
- 2.2.1.1
- 2.2.1.6
- 2.2.1.7
- 2.3.1.1
- 2.3.1.1.17
- 2.3.1.1.2
- 2.3.1.1.28
- 2.3.1.1.57|2.7.7.23
- 2.3.1.1.68

- 2.3.1.1.8
- 2.3.1.2.8
- 2.3.1.2.9
- 2.3.1.3.0
- 2.3.1.3.7
- 2.3.1.4
- 2.3.1.4.1
- 2.3.1.4.8
- 2.3.1.5
- 2.3.1.5.4
- 2.3.1.5.7
- 2.3.1.6
- 2.3.1.7
- 2.3.1.7.4
- 2.3.1.8
- 2.3.1.8.5
- 2.3.1.9
- 2.3.1.9.7
- 2.3.3.1
- 2.3.3.1.0
- 2.3.3.9
- 2.4.1.1
- 2.5.1.1.8
- 2.5.1.1.8|5.2.1.2
- 2.5.1.4.7
- 2.5.1.4.7|2.5.1.65|4.2.1.22
- 2.5.1.6
- 2.5.1.6.1
- 2.5.1.6.6
- 2.6.1.1
- 2.6.1.1.1
- 2.6.1.1.1,2.6.1.1.7
- 2.6.1.1.3
- 2.6.1.1.9
- 2.6.1.1.9|2.6.1.2.2
- 2.6.1.2.1
- 2.6.1.3.0
- 2.6.1.3.6
- 2.6.1.4.2
- 2.6.1.4.4|2.6.1.5.1
- 2.6.1.5.2
- 2.6.1.5.7
- 2.6.1.6.2
- 2.6.1.7,2.6.1.3.9
- 2.6.1.9

- 2.7.1.3.3
- 2.7.1.3.5
- 2.7.2.4
- 2.7.7.2.3
- 2.7.7.3
- 2.7.7.4.8
- 2.7.7.4.8|3.4.2.1.9.1
- 2.8.1.1
- 2.8.1.7
- 2.8.1.7|4.4.1.1.6
- 2.8.3.1.6
- 2.8.4.1

Supplement: Additional file 4 — Figure S2. The SOM labeled with the EC numbers of transferases. File “FigS2.pdf” contains results of the SOM of transferases. The 1,444 RDFs were mapped onto a [40, 19]-sized rectangular lattice, where each color of the node shows the major EC number in a node. The details of catalytic sites mapped onto the SOM were described in Table S2. [file 1472-6807-12-5-S4.pdf]

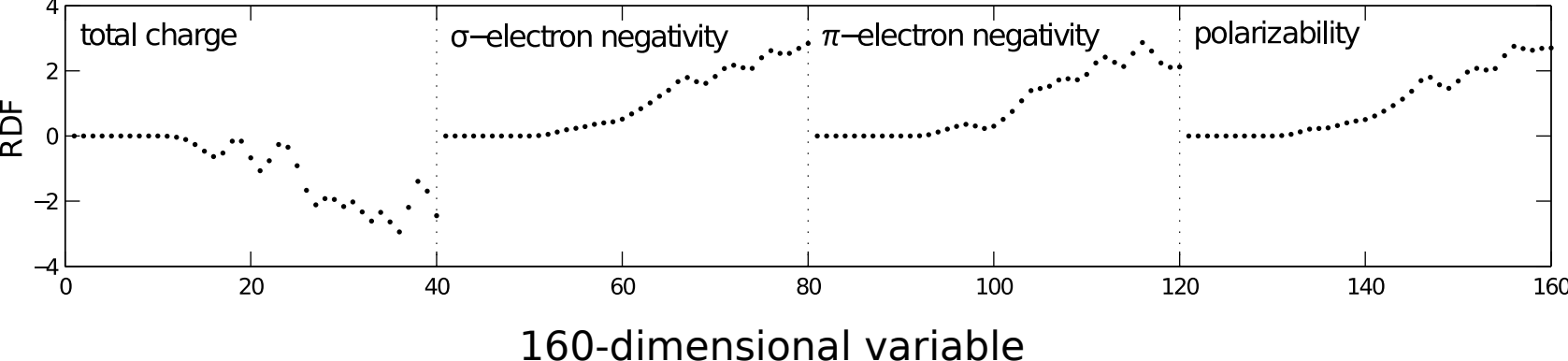

Supplement: Additional file 7 — Figure S3. An example of an RDF. File “FigS3.pdf” contains an example of an RDF for the total charge, σ-electronegativity, π-electronegativity and effective atom polarizability, which constitute a 160-dimensional variable as a feature vector. [file 1472-6807-12-5-S7.pdf]

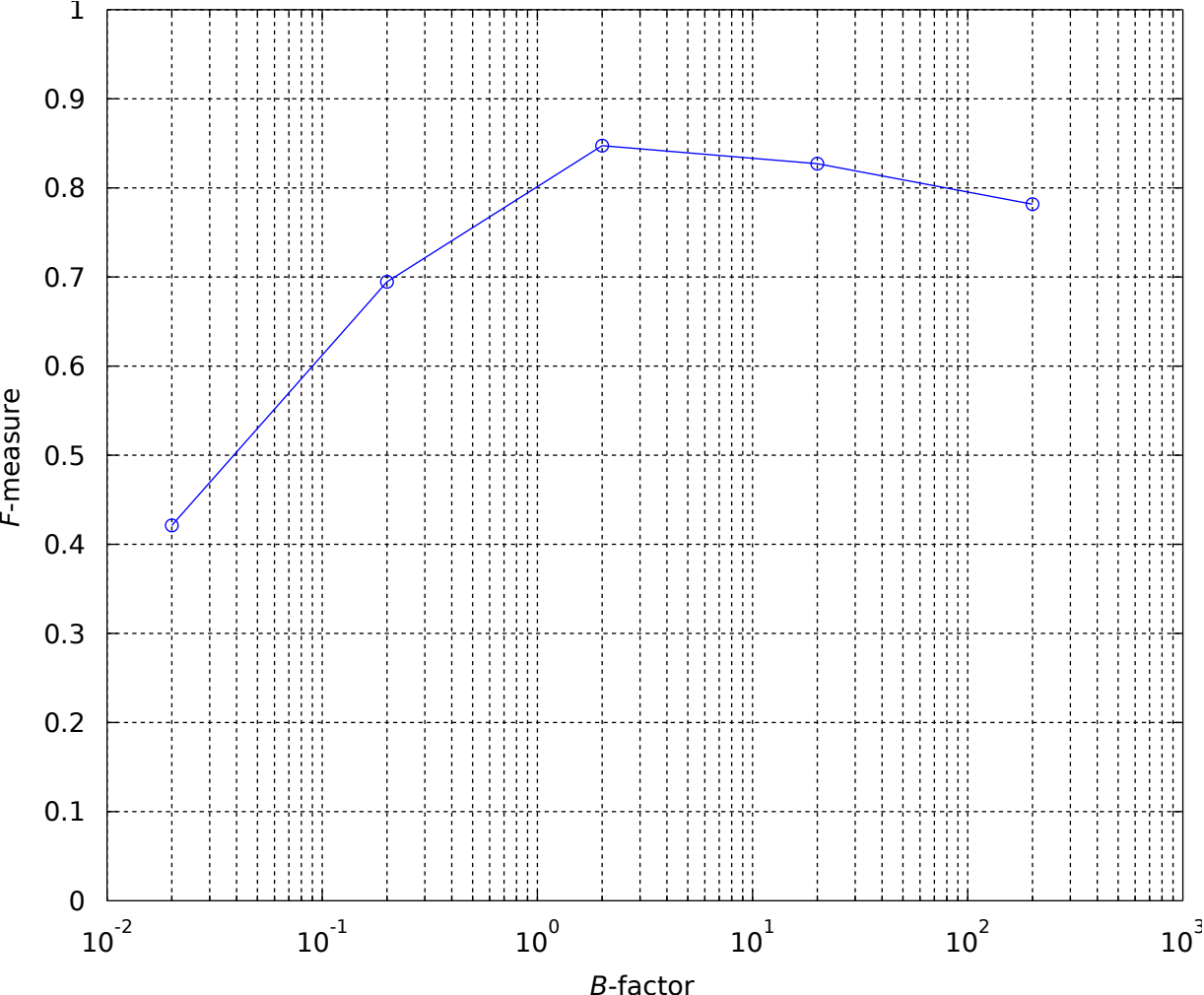

Supplement: Additional file 8 — Figure S4. Robustness of functional classification to conformational change. File “FigS4.pdf” contains the performance of the SOM clustering for the EC numbers as a B-factor in the RDFs is varied. The large B-factor in the RDFs corresponds to conformational change. The F-measure indicates the robustness of the classification performance. [file 1472-6807-12-5-S8.pdf]
